# Hybrid repair of aberrant right subclavian artery with aortic dissection caused by Kommerell diverticulum

**DOI:** 10.1186/s12872-021-02340-8

**Published:** 2021-11-22

**Authors:** Tieyan Li, Lin Zou, Yunzhen Feng, Guoliang Fan, Yuanfeng Xin

**Affiliations:** 1 Department of Cardiovascular Surgery, Shanghai East Hospital, Tongji University School of Medicine, Shanghai, 200120 People’s Republic of China; 2 Department of Endocrinology, Shanghai Pudong New Area Gongli Hospital, Shanghai, 200135 People’s Republic of China; 3 Shanghai Engineering Research Center of Artificial Heart and Heart Failure Medicine, Shanghai, 200120 People’s Republic of China

**Keywords:** Hybrid repair, Kommerell diverticulum, Aortic dissection

## Abstract

**Background:**

Aberrant right subclavian artery (ARSA) with associated Kommerell diverticulum (KD) is a rare congenital aortic disease. KD patients have a high risk of rupture, dissection, and compression of adjacent structures. Although several treatment options have been proposed (traditional surgery, hybrid operation, and endovascular intervention), a consensus regarding optimal surgical management has not yet been established.

**Case presentation:**

A case of successful hybrid repair of distal aortic arch dissection aneurysm by dissecting KD and ARSA with debranching of right and left common carotid arteries, left subclavian artery, and stent grafting was presented.

**Conclusions:**

The hybrid operation is suitable for elderly patients or those with high risks. Along with intervention, the hybrid operation needs to be developed as a minimally invasive method.

## Background

Kommerell diverticulum (KD) is a rare vascular malformation. When the right subclavian artery appears, the fourth arterial arch is enlarged during embryonic development. The incidence of the right subclavian artery accompanied by the left arterial arch is 0.5–2.0%, while that by the right arterial arch is 0.05% [1]. The aortic dissection (AD) and rupture are attributed to the abnormal KD tissue. The treatment methods of the aortic dissection caused by KD include traditional surgery, intervention, and hybrid surgery. Herein, we summarized a case report of hybrid surgery and a 6-month postoperative follow-up on the treatment of KD-induced AD.

## Case presentation

### Case

**Fig. 1:**
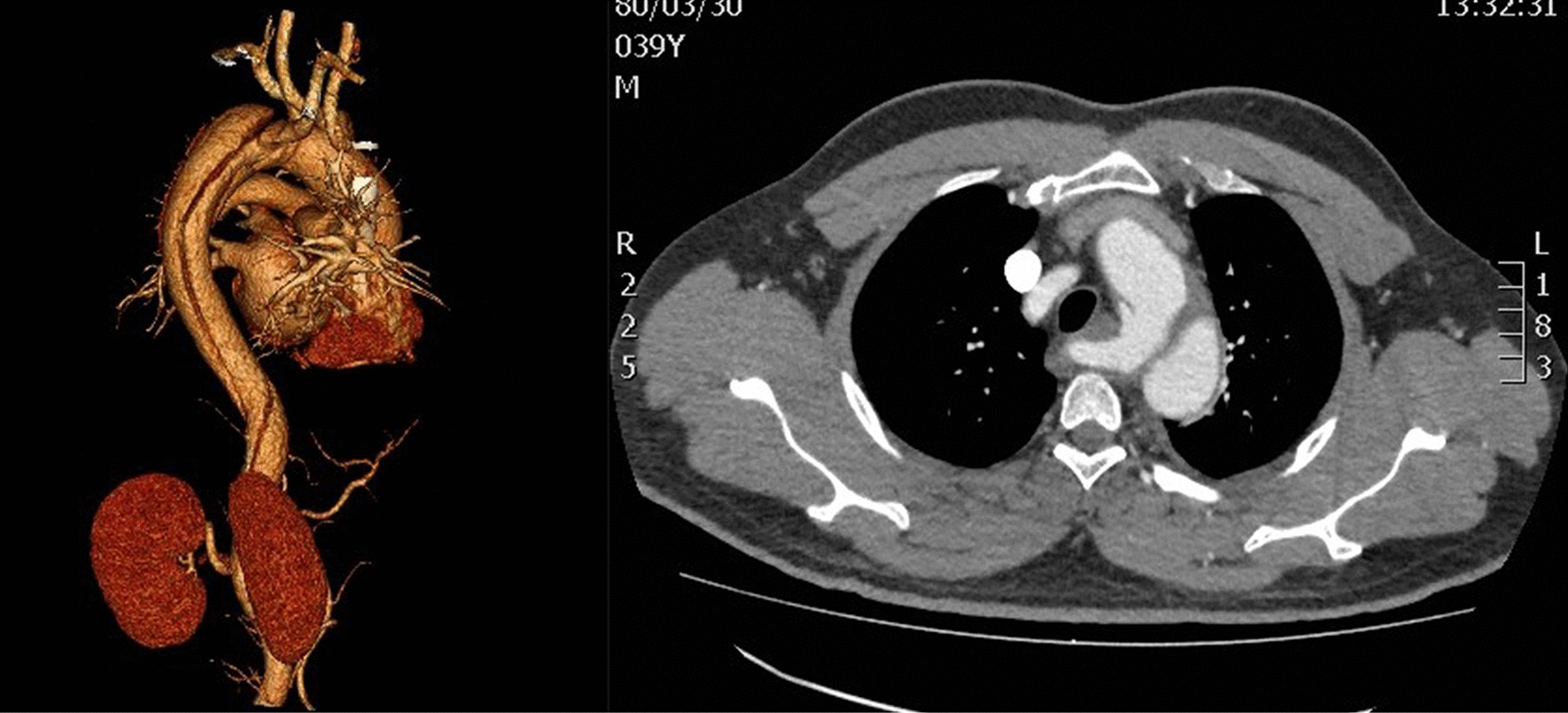
Before the operation

A 39-year-old male patient presented hypertension and was admitted to the Emergency Department with back pain for 5 h but without abdominal pain. The blood pressure was 189/120 mmHg, and the heart rate was 120–150 beats/min (bpm) at the time if admission. The computed tomography angiography (CTA) showed AD (type B) at the origin of KD (Fig. 1), and the original arterial supplied blood to the superior mesenteric artery and renal artery. The celiac artery was fed by AD. The Emergency Department doctor administered beta-blocker, morphine, and sodium nitroprusside. Then, the patient was transferred to the Cardiac Surgery Department.

## Operation

**Fig. 2:**
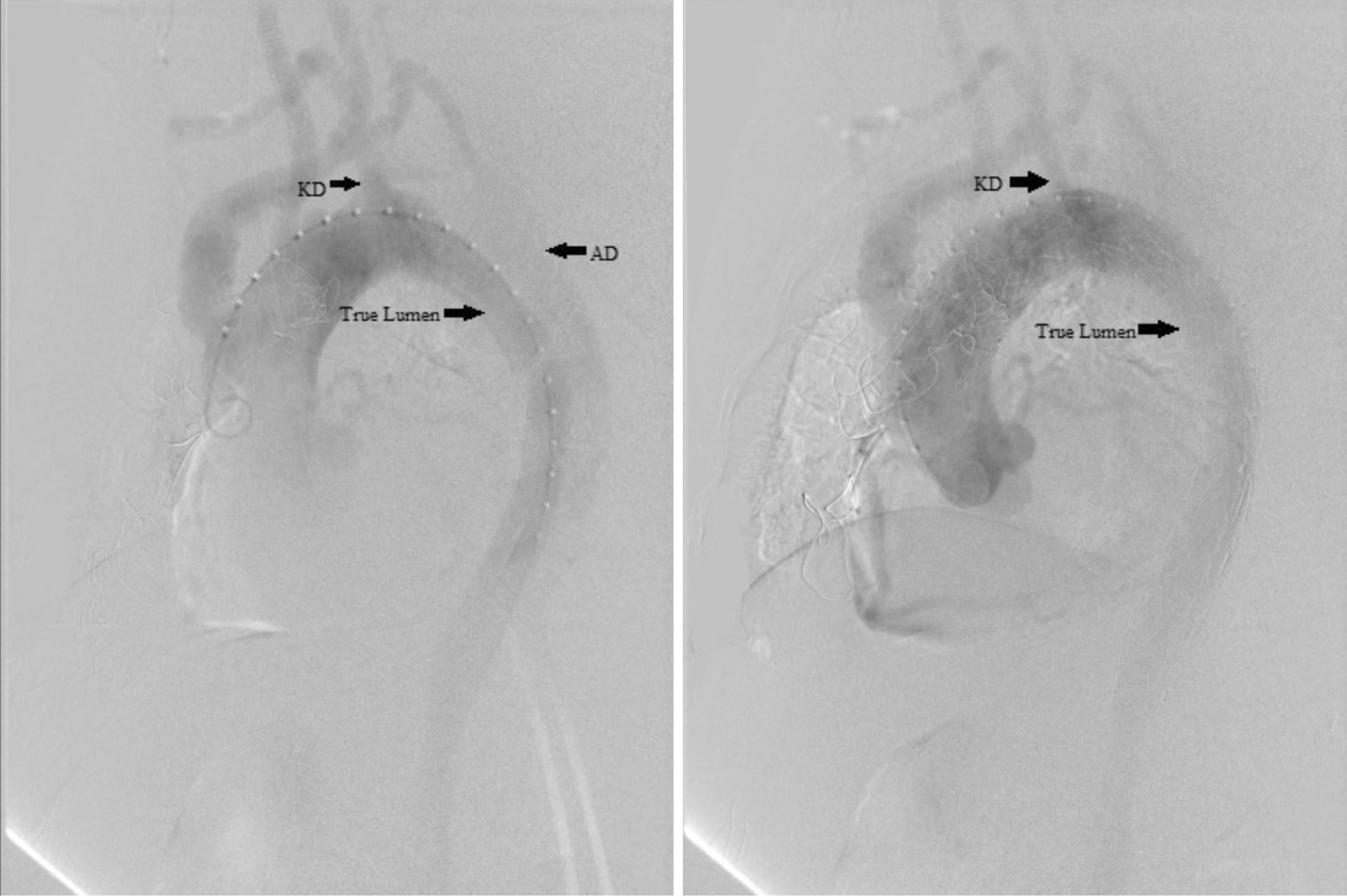
Radiography images during the operation

**Fig. 3:**
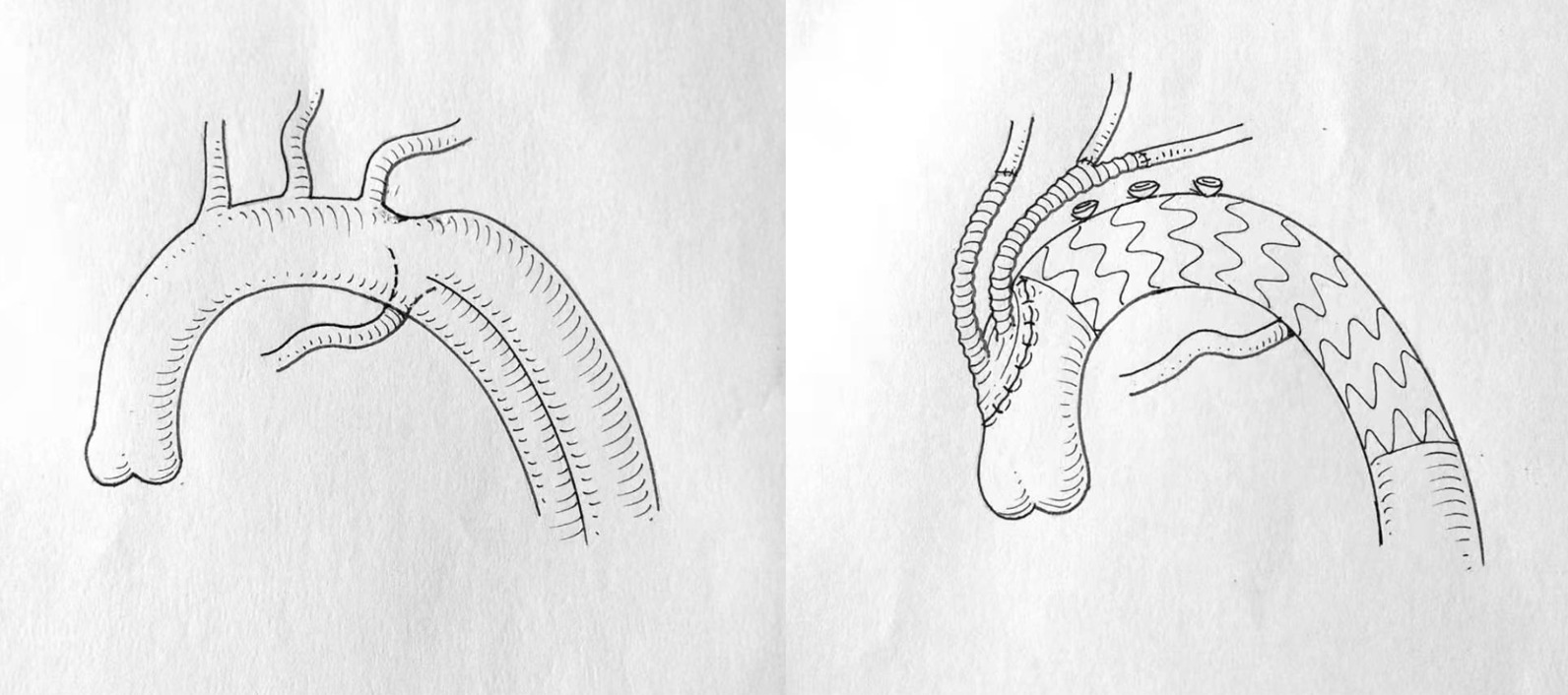
Schematic illustration of the anatomy and the operation. (Our own hand-drawn draft)

The right femoral artery was separated and backed up for cardiopulmonary bypass (CPB) during the operation. The right and left common carotid arteries and the left subclavian artery was dissected through the median incision. The ascending aorta was blocked partially by an occlusion aortic clamp. Then, a 26-mm vascular prosthesis with two 8-mm branches was sutured to the side of the artery using 4-0 prolene. One branch of the artificial vessel was connected to the right common carotid artery, while the other was sutured to the left subclavian artery end-to-end and to the left common carotid artery side-to-end. Subsequently, the intervention was conducted by puncturing a 6 F sheath into the right femoral artery using Seldinger’s method. A super-smooth guidewire was placed into the abdominal aorta through the sheath. The pigtail catheter was inserted into the artery along the guidewire. The radiography image ensured that the catheter was in the true lumen. Then, the catheter with the guidewire was delivered to the ascending aorta. The radiography captured images under the following parameters: left anterior oblique 65°, contrast agent volume 20 mL, flow speed 25 mL/s, and pressure 400 Pa to measure the dissection position and the starting point of KD. Subsequently, two aortic stents were released (Fig. 2). The first one had 38–32 mm width and 160 mm length fixed next to the anastomosis of the thoracic artery and artificial vessel. The second stent was 36–30 mm wide and 160 mm long and fixed partially inside the first one that covered the AD. The final radiography image showed a well-shaped stent without any leak between the stents and vessel (Fig. 3). Next, we used 5-0 prolene to suture the femoral artery. The blood pressure was measured in the femoral and dorsal foot arteries. The pressure of the left and radial arteries was 134/76 and 117/56 mmHg, respectively. Since the difference in the pressure between the left and right artery was < 40 mmHg, and hence, the reconstruction of the right Kommerell artery was rejected.

## Results

**Fig. 4:**
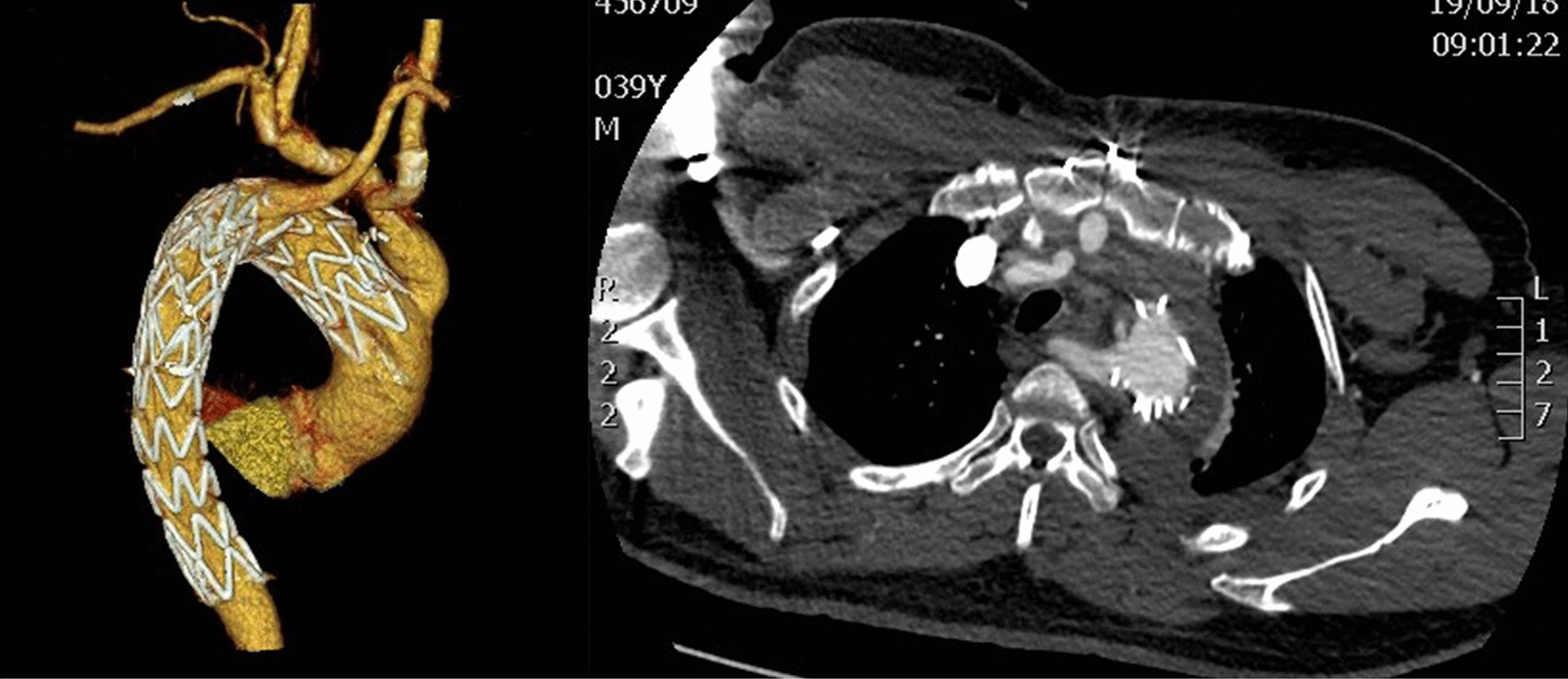
Before discharge from the hospital

**Fig. 5:**
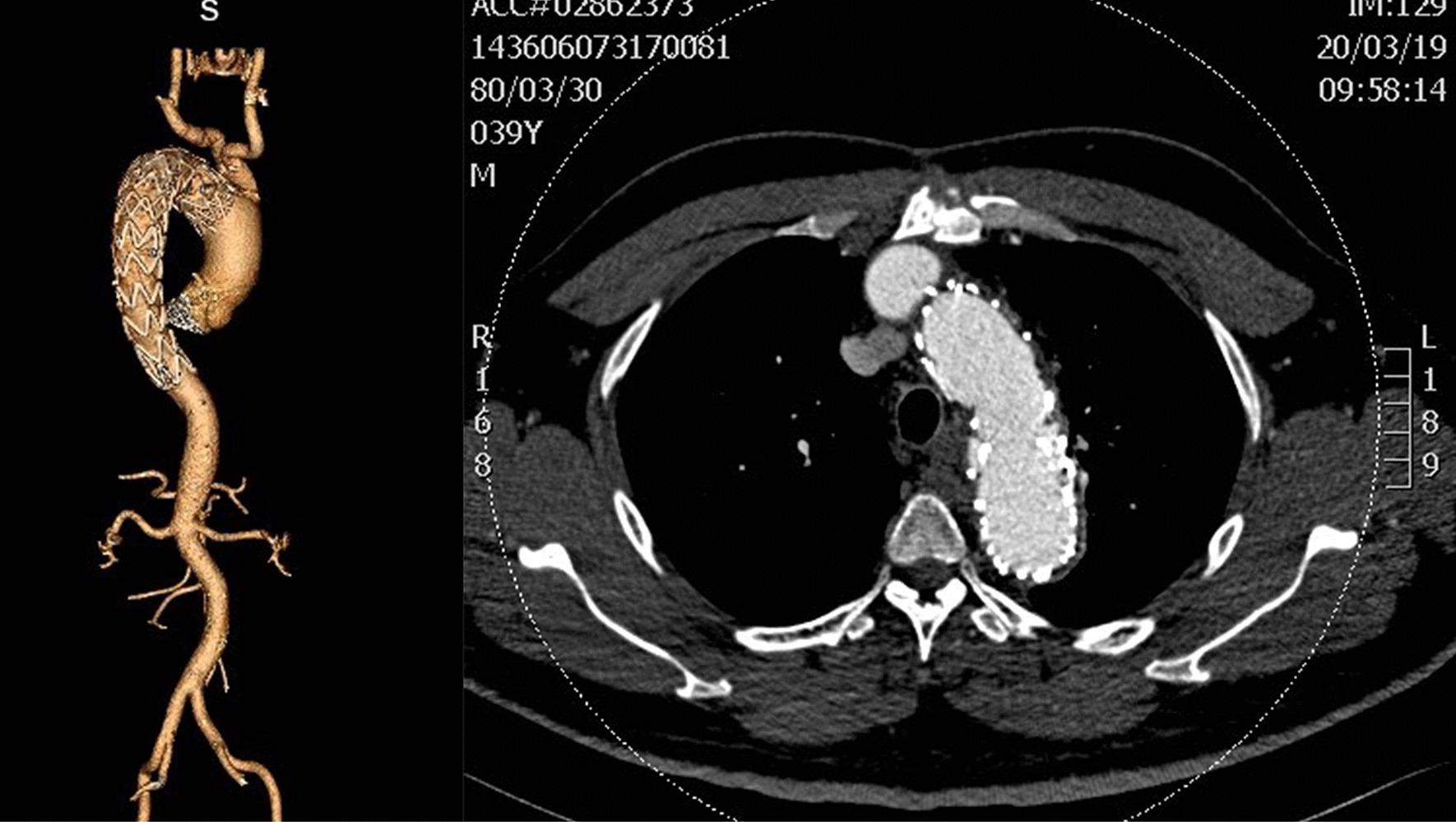
6-month follow-up results

The total operation time was 4 h without CPB. The intubation of the patient was removed after 7 h, but he stayed in the intensive care unit (ICU) for 1 day. The whole therapy process did not involve any blood transfusion. Before discharge, the CTA of the patient showed an open aortic lumen, and the thoracic aortic false cavity was closed, while the right subclavian artery was 8 mm wide (Fig. 4). The abdominal aorta presented AD. However, after 6 months, the abdominal aortic false cavity closed, and the KD disappeared (Fig. 5). The follow-up revealed that all the thoracic and abdominal arterial branches are supplied blood from the true lumen. The difference in the pressure between the two upper limbs was < 10 mmHg.

## Discussion and conclusions

Typically, KD is in a deep position and diagnosed when the diverticulum presses the esophagus or trachea. Previous studies have shown that the incidence of AD in the KD patient is 40–53%, which is higher than that in normal individuals [2]. Based on the site of hemodynamics, the artery receives shear stress, which eases the AD [3]. From a pathological perspective, medial cystic necrosis is common in KD patients, especially in those with atherosclerosis. The weak vascular tissue regresses to the aneurysm, even to AD [4]. The aortic breakage of KD patients often appears near the breach of the diverticulum. Because of the long distance between the breakage and the ascending artery, the artery tears in reverse to the aortic arch. This phenomenon also explains the frequency of Type B than Type A AD. In this case report, the patient presented hypertension history, and hence, the blood pressure was not controlled adequately.

The surgical indication of KD is controversial. Backer et al. suggested that the operation should be conducted when the diameter of the diverticulum is 1.5-fold that of the subclavian artery [5]. Ota et al. proposed that the surgical indication should be considered when the diameter of KD is > 5 cm, and the diverticulum suppresses the mediastinum [6]. According to Cina et al., the difficulty of the surgery depends on the diameter of KD. Thus, intervention is essential when the diameter of the diverticulum is > 3 cm [7].

Furthermore, the surgical indication is definite when KD leads to AD. The operation methods include the traditional surgical operation, thoracic endovascular aortic repair (TEVAR), and hybrid operation. Currently, there is a lack of unified opinion on the operation methods, especially for type B AD caused by KD. If the range of the AD is limited, traditional surgery can be considered. Since the median incision does not provide adequate exposure, the lateral thoracic incision is also needed. The bigger the injury and the deeper the hypothermia, the perioperative mortality and the complication reaches up to 15% [8]. Along with the development of the intervention, TEVAR is a minimally invasive treatment. However, it may cause severe complications because the main branches of the artery are isolated. Although the tailored aortic stent can reduce complications, it is not suitable for emergency operations. The chimney aortic stent is another method, but the endoleak of the stent is about 22% due to the poor compliance of the chimney [9]. Thus, it could be deemed that TEVAR is suitable for elderly patients or those intolerable to a surgery. The hybrid operation consists of the surgical method and TEVAR, which comprises a safe treatment for KD patients with Type B AD. Previous studies have shown that the Z2 zone is the ideal anchor for the aortic stent, while some studies indicate the Z0 as the safe zone [10]. If the aortic atherosclerosis is severe or the aortic calcification is extensive, especially along the aortic arch, the ideal anchor for the aortic stent should be the Z2 or Z3 zone; otherwise, the Z0 zone may be the ideal anchor that shapes the aortic lumen adequately. Herein, we chose the Z0 zone as the anchor in this case. Therefore, the aortic stent had sufficient support area in the artery [11]. The second stent overlapped the first and encompassed that branch of KD. The whole operation process was monitored by transesophageal echocardiography (TOE) that can help the surgeon check the cardiac function and the position of AD. The 6-month follow-up after the operation revealed that the false aortic cavity is closed. The main branches of the abdominal artery are supplied blood via the true artery. Next, we used the debranch of the artificial vessel to rebuild the three main branches of the aortic arch. Thus, it could be stated that the advantage of hybrid operation is minimal injury. The connection between the ascending artery and the artificial vessel is implied without CPB. However, the operation does not require deep hypothermia. Surgical hemostasis is much easier than the traditional aortic operation. The surgery provides an anchor zone that can reduce the incidence of type I endoleak. The debranch of the artificial vessel ensures sufficient blood supply to the brain and limbs. The 6-month outpatient follow-up results did not show any significant difference between the pressures of the upper limbs.

Taken together, KD coincided with aortic dissection is a rare disease. The hybrid operation is suitable for elderly patients or those with high risks. Along with intervention, the hybrid operation needs to be developed as a minimally invasive method.

## Data Availability

The dataset supporting the results of this study is included in the article.

## Abbreviations

CPB: Cardiopulmonary bypass
CTA: Computed tomography angiography
ICU: Intensive care unit
KD: Kommerell diverticulum
TEVAR: Thoracic endovascular aortic repair
